# Targeted In Situ Gene Correction of Dysfunctional *APOE* Alleles to Produce Atheroprotective Plasma ApoE3 Protein

**DOI:** 10.1155/2012/148796

**Published:** 2012-05-07

**Authors:** Ioannis Papaioannou, J. Paul Simons, James S. Owen

**Affiliations:** Division of Medicine, UCL Medical School, Royal Free Campus, Rowland Hill Street, London NW3 2PF, UK

## Abstract

Cardiovascular disease is the leading worldwide cause of death. Apolipoprotein E (ApoE) is a 34-kDa circulating glycoprotein, secreted by the liver and macrophages with pleiotropic antiatherogenic functions and hence a candidate to treat hypercholesterolaemia and atherosclerosis. Here, we describe atheroprotective properties of ApoE, though also potential proatherogenic actions, and the prevalence of dysfunctional isoforms, outline conventional gene transfer strategies, and then focus on gene correction therapeutics that can repair defective *APOE* alleles. In particular, we discuss the possibility and potential benefit of applying in combination two technical advances to repair aberrant *APOE* genes: (i) an engineered endonuclease to introduce a double-strand break (DSB) in exon 4, which contains the common, but dysfunctional, **ε**2 and **ε**4 alleles; (ii) an efficient and selectable template for homologous recombination (HR) repair, namely, an adeno-associated viral (AAV) vector, which harbours wild-type *APOE* sequence. This technology is applicable ex vivo, for example to target haematopoietic or induced pluripotent stem cells, and also for in vivo hepatic gene targeting. It is to be hoped that such emerging technology will eventually translate to patient therapy to reduce CVD risk.

## 1. Introduction

Deaths from cardiovascular disease (CVD), which encompasses ischaemic heart disease, stroke, and peripheral vascular disease, total about 17 million per year worldwide, almost one-third of the total [1]. Notably, over 80% of CVD deaths are in low- and middle-income countries; it is not a disease confined to developed countries. Atherosclerosis is a progressive inflammatory response to complex interactions between cell types endogenous to the arterial wall, monocytes, lymphocytes and platelets from blood, and circulating lipoproteins [2]. Early atherosclerotic lesions are lipid streaks, characterized by cholesterol-engorged foam cells within the vascular endothelium. Foam cells originate from blood monocyte macrophages, which are recruited into the arterial intima by upregulated adhesion molecules on activated endothelium. There via unregulated scavenger receptors they relentlessly ingest oxidized low-density lipoprotein (LDL) or triglyceride-depleted (but cholesterol-containing) remnant lipoprotein particles. Failure to resolve the inflammation eventually leads to smooth muscle cells infiltration, which proliferate within the intima to foster development of established lesions.

A significant risk factor for CVD is increased plasma LDL. This is susceptible to oxidation and is the main source of the cholesterol which deposits in arteries during endothelium injury. The statin class of drugs helps prevent early morbidity or death by lowering plasma LDL. However, statins do not rectify low levels of atheroprotective high-density lipoproteins (HDLs), which is an important independent risk factor because of its role in removing excess cholesterol from arterial walls [3, 4]. Although aggressive high-dose statins are reported to successfully regress atherosclerotic plaque with the goal of reducing morbidity and mortality, the incidence of side effects (liver damage, rhabdomyolysis, and cancer) increases and the therapeutic value remains contentious. Similarly, the value of statin treatment in combination with ezetimibe, a cholesterol-absorption inhibitor which further decreases LDL cholesterol by 15–20%, has been questioned as no significant reductions in intima-media thickness were observed [5]. Alternative strategies to combat occlusive CVD are still urgently needed for many patients [3, 4, 6].

These include drugs to raise HDL levels, such as niacin, inhibitors of cholesteryl ester transfer protein (CETP), and RVX-208, a small quinazoline-family member which upregulates ApoAI, the main HDL protein constituent [4–9]. Gene-based therapies are also receiving considerable attention, including antisense oligonucleotides (ASO) to target LDL's structural protein ApoB100 [10]. Gene silencing of proprotein convertase subtilisin/kexin type 9 (PCSK9) also lowers LDL indirectly. Secreted PCSK9 binds to the LDL receptor (LDLR) and accelerates its degradation, and hence prolonging LDLR activity by PCSK9 inhibition lowers plasma LDL levels [11, 12]. HDL supplementation agents are also in clinical use, including infusion of synthetic or plasma-purified ApoAI and ApoAI mimetic peptides [13]. Gene addition has also been used to boost plasma ApoAI and HDL in preclinical studies using adenovirus and adeno-associated virus (AAV) vectors for delivery [14, 15].

## 2. The ApoE Gene and Protein

**Figure 1:**
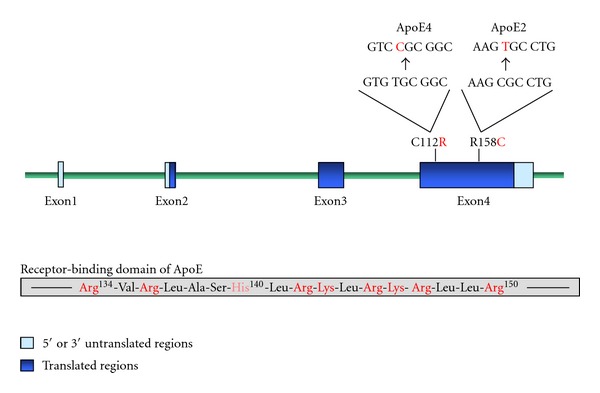
Structure of the human *APOE *gene. The *APOE* gene has 4 exons, comprising 44, 66, 193, and 860 nucleotides, with over 80% of the protein coded for by exon 4. Exon 1 contains 5′ untranslated sequence, while the translated sequence in exon 2 codes for most of the 18 residue signal peptide. As indicated, exon 4 contains the two common disease-associated SNPs: a T→C point mutation produces ApoE4 (Cys112Arg), while C→T mutation gives ApoE2 (Arg158Cys). The wild-type sequence is ApoE3 (Cys112, Arg158). Also shown in the lower part of the figure is the positively charged arginine- and lysine-rich *α*-helical segment (residues 134–150) which recognizes the LDL receptor.

Apolipoprotein E (ApoE) is a 34-kDa polymorphic glycoprotein largely secreted by liver (~90%; 40–60 *μ*g/mL plasma), although other tissues particularly macrophages contribute [16]. The human gene is located on chromosome 19, at the 5′ end of a 50 kb gene cluster comprising ApoCI, an ApoCI pseudogene, ApoCII, and ApoCIV [3]. Like many of the soluble apolipoproteins, the ApoE gene has four exons separated by three introns and is synthesized and released via the canonical pathway (Figure 1). The primary translation product is 317 residues containing an N-terminal 18 amino acid (a.a.) signal peptide to direct the growing ApoE polypeptide to the endoplasmic reticulum. Prior to secretion, ApoE undergoes* O-*linked glycosylation in the Golgi, principally at Thr^194^ although carbohydrate chains containing sialic acid are also present on Ser^290^.

ApoE protein comprises an N-terminal domain (1–191 residues) of four amphipathic *α*-helices linked to the C-terminus by a protease-sensitive loop [17]. An arginine- and lysine-rich segment in helix-4 (residues 134–150; Figure 1) contains the recognition site for the LDLR and LDL receptor-related protein (LRP). ApoE contains two heparin-binding sites, one within the receptor-binding domain and the other within the C-terminus (residues 243–272). There are three common isoforms of ApoE, termed E2, E3, and E4, which are the products of three alleles (*ε*2, *ε*3, and *ε*4) at a single gene locus [16]. The ApoE3 (Cys^112^, Arg^158^) is considered the parent form, while ApoE4 (Arg^112^, Arg^158^) and ApoE2 (Cys^112^, Cys^158^) arise from genetic point mutations and are variants (Figure 1). There is considerable allelic variation in different populations, but, in Europe, the relative frequencies are approximately 0.77, 0.15, and 0.08 for *ε*3, *ε*4, and *ε*2 [18], giving in order of occurrence E3/3, E4/3, E3/2, E4/4, E4/2, and E2/2 phenotypes.

The rarest variant ApoE2 is the cause of Type III hyperlipoproteinaemia in a small proportion (~5%) of *ε*2/2 individuals, which confers a markedly increased risk of CVD [19, 20], and the allele is also tentatively linked to intracerebral haemorrhage [21]. Nevertheless, *ε*2 carriers have reduced levels of total and LDL cholesterol (see Section 3), and their risk of coronary heart disease is 20% lower than people with the common *ε*3/3 genotype [22], ApoE4 associates with incidence of ischaemic stroke and subarachnoid haemorrhage [21] and produces an adverse lipoprotein profile, with increased LDL and a slight reduction in HDL [22]. In 1996, a meta-analysis of 14 studies found that carriers of the *ε*4 allele had an increased risk of coronary heart disease [23], and this was confirmed in a subsequent meta-analysis of 48 studies involving 15,942 disease cases, which concluded the risk was 42% higher compared to *ε*3/3 carriers [24]. Moreover, the LDL cholesterol level is 30% lower in people with the *ε*2/2 genotype than with *ε*4/4, a reduction comparable to that achieved with statins. Nevertheless, this supposition of risk for *ε*4 carriers is contentious; an updated meta-analysis, which focused on studies recruiting large numbers of participants to reduce publication bias, reported only a modest increase of risk [22]. On the other hand, *APOE*: environment interactions on CVD risk are attracting increased attention [25]; the *ε*4 allele potentiates the risk of CVD from physical inactivity and also from smoking, though mainly in women [26]. In addition, *ε*4 carriers have a lower life expectancy, a finding reflecting their predisposition to neurodegenerative disease as well as CVD [27, 28].

As detailed in Sections 3 and 4, and independently of genotype, ApoE has a plethora of actions to inhibit atherogenesis. Most information has come from studies in vitro or in mice, and, although many mechanisms are ill-understood, the weight of evidence strongly suggests that ApoE is atheroprotective. But there is a final twist to the tale: plasma ApoE levels positively correlate with CVD mortality [29], leaving several unanswered questions concerning human ApoE biology in health and disease [30].

## 3. ApoE and Lipid-Related Atheroprotection

ApoE plays an essential role in the metabolism of dietary lipids, which enter the circulation as large triglyceride-rich chylomicron particles. Following lipolysis and uptake of energy-rich monoglycerides and fatty acids, cholesterol-containing and potentially atherogenic remnant particles are left, which depend on ApoE for rapid hepatic clearance via pathways involving LRP, the LDLR and heparan sulphated proteoglycans (HSPG) [31]. ApoE4 has marginally greater receptor-binding capability than ApoE3, but ApoE2 is defective with only 2% and 40% binding activity to the LDLR and LRP, respectively. Although the ApoE2 isoform has an amino acid substitution (Arg158Cys) outside of the 134–150 receptor-binding domain (Figure 1), this has a major disruptive influence. In ApoE3, a salt bridge is formed between Arg158 and Asp154. However, this interaction is lost in ApoE2, and so Asp154 forms a bridge with Arg150, reducing the positive potential of the binding site to markedly impair its binding affinity [32].

When ordered E2/2, E2/3, E2/4, E3/3, E3/4, and E4/4, there is a stepwise increase in plasma LDL cholesterol [22, 33]. One explanation is that this reflects up-or downregulation of the hepatic LDLR due to changes in cholesterol delivery by ApoE2- or ApoE4-containing remnant particles, respectively. Thus, a diminished cholesterol supply by ApoE2 would increase hepatic LDLR numbers to lower plasma LDL; additionally, reduced competition between ApoE2-remnants and LDL for binding by the LDLR would result in accelerated LDL clearance [19]. The higher binding affinity of ApoE4-containing lipoproteins for the LDLR would have opposite effects and so raise LDL levels. Nevertheless, the mechanism is more complex since conversion of VLDL to LDL is impaired by ApoE2, which reduces the amount of LDL formed [19]. 

ApoE also contributes to “reverse cholesterol transport,” the antiatherogenic HDL-dependent pathway by which excess cholesterol in peripheral tissues, including arteries, is brought to the liver for biliary excretion. Such regulation of cellular cholesterol homeostasis, particularly in macrophages, is vital in preventing foam cell formation and atherogenesis. Efficient cellular cholesterol efflux depends on ATP-binding cassette transporters (ABCA1 and ABCG1), but lipid-poor ApoAI- (pre*β*-1 HDL) and ApoE-containing particles (*γ*-LpE) are avid initial cholesterol acceptors. Significantly, the plasma *γ*-LpE fraction from E3/3 subjects sequestered substantially more cellular cholesterol than the fractions in E2/2 and E4/4 plasmas [34]. Additionally, ApoE can activate plasma lecithin-cholesterol acyltransferase (LCAT), CETP, and hepatic lipase (HL), which are all involved in HDL maturation [16]. As macrophages secrete ApoE at lesion sites, it is the dominant acceptor in clearing excess arterial cholesterol. This effect is isoform dependent with ApoE2/2 human monocyte macrophages secreting substantially lower amounts of ApoE compared to E3/3 or E4/4 cells [35], apparently because in macrophages, though not in hepatocytes, cysteine-rich ApoE2 forms dimers and multimers which are bound to LRP and retained in the secretory pathway [36].

These atheroprotective biological functions of ApoE are corroborated by the hyperlipidaemia and atheroma seen in ApoE-deficient (ApoE^−/−^) mice. Normal mice resist atherosclerosis and even when fed a proatherogenic diet develop only immature fatty streak lesions [37]. However, ApoE^−/−^ mice are grossly hypercholesterolaemic on normal chow and spontaneously develop widespread fibroproliferative lesions, which evolve into advanced complex plaques with smooth muscle cell caps and necrotic cores [38]. On the other hand, transgenic animals expressing the common human ApoE isoforms have revealed more subtle aspects of ApoE atheroprotection [37]. For example, early studies in both mice and rabbits showed that overexpression of ApoE3 was detrimental causing hypertriglyceridaemia. Excessive production of hepatic ApoE stimulates VLDL triglyceride synthesis and also inhibits lipoprotein-lipase- (LPL-) mediated lipolysis, largely by displacing ApoCII an essential cofactor for LPL from the VLDL surface [19]. However, phenotypic interpretations and comparison of data from different groups were sometimes confounded because the human *APOE *transgene was inserted into different genomic locations and at varying copy numbers and because endogenous mouse ApoE protein was also present.

These difficulties were mitigated by introduction of gene replacement strategies to generate mice in which the mouse *Apoe *gene was replaced by a human allele. Nevertheless, such mice also have limitations: unlike wild-type mice, ApoE3 knock-in animals were susceptible to dietary-induced hypercholesterolaemia and atherosclerosis, apparently because human ApoE3 has low receptor-binding affinity and is less efficient at clearing remnant particles [39]. Moreover, type III hyperlipoproteinaemia in humans homozygous for the *ε*2 allele is a recessive condition, whereas a dominant inheritance is seen in ApoE2 knock-in mice [40]. While this may also reflect binding differences between human and mouse receptors, it should be noted that ~80% of liver-derived mouse VLDL is ApoB48-containing particles, and, hence, mice depend much more on ApoE for remnant clearance than do humans. Interestingly, many of the rare ApoE variants with amino acid substitutions within the receptor-binding region do associate with dominant type III hyperlipoproteinaemia even though most have adequate receptor-binding activity compared to ApoE2 [19]. Studies In vitro studies [41], and also in ApoE2 and ApoE (Arg142Cys) transgenic mice [19] suggest that recessive ApoE2 retains HSPG binding, allowing remnant clearance via this alternative pathway [19], whereas the dominant ApoE variants have negligible binding which translates to an overall clearance rate lower than that of ApoE2 [31].

## 4. ApoE and Lipid-Independent Atheroprotection

**Figure 2:**
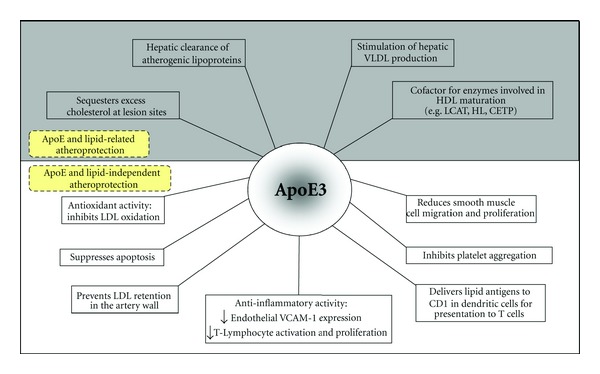
Proposed atheroprotective functions of ApoE3. The antiatherogenic properties of ApoE3 are divided into those related to its role in lipid metabolism (shaded area) and those independent (unshaded area).

It is now recognized that ApoE fulfils several biological functions unrelated to lipid transport and that these make significant contributions to its antiatherogenic activity [16, 42–44] (Figure 2). Most are anti-inflammatory in nature and include the early observation that ApoE restricts T-cell activation and proliferation [45]. Later, our own studies showed that cell-derived ApoE inhibited platelet aggregation and also the expression of vascular cell adhesion molecule 1 (VCAM-1) on endothelial cells, actions mediated by the common mechanism of ApoE interaction with the cell-surface receptor, LRP8 (ApoER2) to activate nitric oxide synthase (NOSIII) and release NO [46, 47]. Additional data suggest that ApoE stimulates tyrosine phosphorylation of LRP8 to initiate PI3 kinase signalling and activation of NOSIII [48]. Induction of smooth muscle cell migration and proliferation by oxidized LDL or platelet-derived growth factor are also suppressed by ApoE [49], while subendothelial retention of LDL, an early proatherogenic event [50], is impeded by the presence of ApoE, which additionally regulates the availability of cytokines and growth factors within the pericellular proteoglycan matrix [51]. There is clear evidence that ApoE protects cells and lipoproteins against lipid oxidation and other oxidative stresses in an isoform-dependent manner, although findings can vary with the experimental system used. In cell-free systems, metal-induced oxidation is inhibited by ApoE in an allele-specific manner (E2>E3>E4), largely because it sequesters metal ions [52–54]. However, when LDL was incubated with transfected macrophages secreting equal amounts of ApoE, the ApoE3 and ApoE4 isoforms provided greater protection against oxidation than ApoE2 [54]. Transfected mouse peritoneal macrophages have also been shown to have anti-inflammatory properties with ApoE3-expressing cells secreting lower levels of the proinflammatory cytokines IL-6 and TNF-*α* than ApoE2 and ApoE4 cells [55].

Studies in vivo using ApoE^−/−^ mice have also given important clues to possible atheroprotective actions of ApoE. For example, lipoproteins from ApoE-deficient mice are more oxidized and prone to oxidation than those from control mice [56], while clearance of apoptotic cell remnants is reduced in the absence of ApoE [57]. An anti-inflammatory role for ApoE in dampening inflammation induced by lipopolysaccharide (LPS) or bacteria is postulated based on the increased susceptibility of ApoE^−/−^ mice compared to control animals when challenged with such pathogens [58–60]. By contrast, a specific proinflammatory role for ApoE was suggested by van den Elzen and colleagues who showed that ApoE delivers lipid antigens to CD1 endosomal compartments of antigen-presenting dendritic cells, most likely via LDLR endocytosis, to stimulate natural killer T-cells [61]. This activation was lost from ApoE-depleted human serum and also drastically reduced in ApoE-deficient mice challenged with an exogenous lipid antigen. The authors also speculated that ApoE might use this pathway to deliver self-lipid antigens and exacerbate atherosclerosis, although such removal of antigenic lipids might also be atheroprotective. A full description of the cross-talk between ApoE and cytokines, including modulation of inflammatory and immune responses and isoform dependency, is beyond the scope of this paper but was recently reviewed by Zhang and colleagues [44]. Moreover, the (patho)physiological importance of such lipid-independent actions of ApoE is highlighted by studies in which low-level expression continues to provide atheroprotection in ApoE^−/−^ animals, despite no change in their hyperlipidaemia [42, 62]. Significantly, Raffai et al. [63] provided compelling evidence that low levels of ApoE protein have the capacity to *regress* preexisting atherosclerotic lesions, independently of lowering plasma cholesterol.

## 5. ApoE Gene-Augmentation Therapeutics

### 5.1. Early Insights from Protein Therapy and Transgenic Mice

Twenty years ago plasma-purified or recombinant ApoE protein were infused into rabbits with genetic or diet-induced hypercholesterolaemia. Plasma cholesterol levels were markedly reduced, while a long-term study halted atherosclerotic plaque development [64]. This preclinical evidence endorsed the *APOE *gene as a candidate for therapeutic manipulation. Similarly, synthetic peptide mimics of the ApoE binding region (based on a dimeric repeat of a.a. 141–155) cleared cholesterol-rich lipoproteins in ApoE^−/−^ mice [65], a strategy which evolved into covalently coupling ApoE 141–150 residues to an 18 a.a. amphipathic helical peptide capable of associating with atherogenic ApoB-containing lipoproteins [66]. Thrice weekly intravenous injection of this dual domain peptide into ApoE^−/−^ mice for 6 weeks reduced plasma cholesterol and atherosclerotic lesions in the aortic sinus [67].

Use of in vivo ApoE-secreting miniorgans substantiates these antiatherogenic actions of ApoE peptides and purified protein. Recombinant ApoE-expressing endothelial cells embedded in Matrigel were injected intradermally into ApoE^−/−^ mice which, 3 months later, had 50% less plasma cholesterol and reduced atherosclerotic plaque [68]. Similarly, implantation of alginate-encapsulated engineered cells into the peritoneum of ApoE^−/−^ mice secreted sufficient ApoE to lower plasma cholesterol and increase atheroprotective HDL [69]. Transgenic mice overexpressing ApoE provide additional evidence for ApoE atheroprotection, as these animals are protected from diet-induced or diabetic hyperlipidemia [70, 71]. Moreover, macrophage-restricted expression of ApoE in transgenic mice [72], or in ApoE^−/−^ mice following transplantation of wild-type bone marrow [73, 74], inhibits atherogenesis.

### 5.2. *ApoE* Gene Transfer Studies

 Gene transfer of ApoE was first reported 16 years ago using recombinant adenovirus (rAd) vectors, which deliver foreign DNA into mammalian liver with near-100% efficiency. High levels of plasma ApoE were obtained following intravenous injection of rAd.ApoE3 into ApoE^−/−^ mice, which reduced plasma cholesterol and slowed aortic atherogenesis [75]. Unfortunately, the therapeutic effect was transient, as these 1st generation vectors triggered a strong T-cell immunological response. By contrast, 2nd generation vectors gave sustained human ApoE expression and largely normalized the lipoprotein profile in ApoE^−/−^ mice throughout the 6 week study [76]. Moreover, in hyperlipidaemic LDLR null mice, hepatic expression of human ApoE3 induced regression of preexisting atherosclerotic lesions without altering plasma lipoprotein levels [77], implying lipid-independent atheroprotective actions (Figure 2). The potential to regress advanced atheroma in older animals was also noted [78], while the low toxicity and low immunogenicity of helper-dependent rAd vectors allowed high stable expression of ApoE and lifelong atheroprotection in ApoE^−/−^ mice [79].

An alternative viral vector to rAd is adeno-associated virus (AAV), which contains a linear single-stranded DNA genome and is now at the forefront of clinical gene therapy trials. Early studies used vectors derived from the common serotype 2, which transduced cells very efficiently in vitro, but unfortunately were largely ineffective in vivo. However, the field was boosted by the isolation of over 10 new serotypes, which led to development of new pseudotyped rAAV vectors that is, capsids of alternative AAV serotypes harbouring the recombinant AAV serotype 2 genome. These had strikingly improved performances in vivo and different tissue tropisms to AAV2/2; for example, rAAV2/8 transduced liver with near-100% efficiency [80–82], while rAAV2/1 [83], rAAV2/6 [84] or rAAV2/7 [85] were effective for skeletal muscle transduction.

This increased serotype efficiency was shown by intravenous (liver-directed) injection of a human ApoE3 rAAV2/8 vector into ApoE^−/−^ mice; normal human levels (50-80 *μ*g/mL) of plasma ApoE were produced [58]. It is proposed that, unlike serotype 2, these different AAV serotypes uncoat rapidly to facilitate annealing of the single-stranded plus and minus rAAV genomes into stable, transcriptionally active double-stranded DNA molecules [86]. Similarly, the recent introduction of self-complementary rAAV (scAAV) vectors, which circumvent the need for annealing rAAV vector genomes, allows a rapid and higher level of transgene expression [87, 88]. For example, expression of ApoE3 from scAAV2/8 using the hepatocyte-specific promoter (LP1) normalized cholesterol levels in male ApoE^−/−^ mice and retarded development of aortic atherosclerosis by 58% [89].

Skeletal muscle, an accessible, stable, and well-vascularized tissue, has also been used for gene transfer of ApoE. Although muscle does not normally secrete ApoE, nonhepatic, nonmacrophage-derived ApoE is known to be atheroprotective [62, 90]. Indeed, intramuscular injection of a plasmid DNA vector expressing human ApoE3 into ApoE^−/−^ mice gave modest but sustained lowering of plasma cholesterol [91], while another study reported reduced xanthoma and atherosclerotic plaque formation [92]. The high purity and low immunogenicity of plasmids, which are expressed episomally making insertional mutagenesis improbable, make them attractive delivery vehicles. Expression can be further increased by electropulsing the injection sites(s) [93]. Additionally, we have injected AAV2/7, AAV2/8, and AAV2/9 vectors expressing human ApoE3 into the tibialis anterior muscles of ApoE^−/−^ mice. The first two vectors were the most effective, producing up to 2 *μ*g ApoE3/mL plasma, and, at 13 weeks, the mice had 50% less plaque lipid in brachiocephalic arteries than AAV2/9-treated animals [94].

## 6. Oligonucleotide-Mediated Gene Editing of the Human *APOE* Gene in Cultured Cells

### 6.1. Background

Gene editing, as described herein, uses short synthetic oligonucleotides to manipulate genomic DNA and introduce small, site-specific changes, typically 1–3 nucleotides, into a selected gene of a living cell. It has potential, therefore, for introducing specific mutations, gain or loss of function, into cell lines or mouse strains, and for studying single-nucleotide polymorphisms (SNPs). Below and in Section 7, we describe some of the strategies used to achieve genotypic modifications. However, it is important to note that, unlike gene transfer technologies, the change is permanent; moreover, existing gene enhancers and promoters, and cell-specific control and context, are retained. These advantages underpin its ultimate goal: the cure of hereditary diseases caused by genetic point mutations or small deletions/insertions. First, we explain why synthetic oligonucleotides, which are relatively inexpensive and offer flexible design and chemistry, remain of value for generating new isogenic cell lines, for example, to investigate differential effects of ApoE isoforms on cellular metabolism. Second in Section 7. we describe the rapid progress being made towards therapeutic applications, for example, the ex vivo or in situ repair of genetic point mutations.

### 6.2. Gene Editing: Early Use of RNA-DNA Oligonucleotides

Conceptually, this technology is founded on the independent observations of Smithies and Capecchi in the 1980s that homologous recombination (HR) between a large exogenous DNA segment and its target sequence in a mammalian genome can mediate genomic modification or repair [95, 96]; it also draws on the application of short synthetic oligonucleotides to repair defective alleles in yeast [97]. The use of hybrid RNA-DNA oligonucleotides (RDOs or chimeraplasts) to introduce site-specific changes of 1–3 nucleotides into mammalian genomes was reported 15 years ago by the Kmiec group [98]. They constructed RDOs to form a double-stranded, hairpin-capped oligonucleotide incorporating a short region of correcting DNA bounded by long stretches of protected 2′-O-methyl RNA. The strong RNA-DNA base pairing was considered to promote strand invasion and annealing to the target (e.g., a genetic point mutation) genomic locus. This high-affinity hybridisation involves both strands of the gene and creates the mismatch at the point mutation, leading to recognition and correction by one or more of the mammalian gene repair pathways. The early studies of Kmiec and colleagues in repairing the sickle cell mutation in a lymphoblastoid cell line [99] and in targeting primary CD34+ cells [100] generated much excitement, but also notable controversy as the reported conversion efficiencies of up to 50% were considered implausible [101]. However, these criticisms were deflected by corroboration from others, including targeting human hepatocyte cells [102, 103] and, in landmark studies, in vivo targeting of the rat *factor IX* gene [104] and correction of the *UGT1A1 *gene mutation in the hyperbilirubinaemic Gunn rat [105].

Based on these successes, our laboratory pioneered the technique in cardiovascular disease, including conversion of dysfunctional ApoE2 [106] and ApoE4 [107] to ApoE3 using a standard 68-mer RDO for targeting. However, the practicality of this emerging methodology was questioned when several groups, our own included, began to report poor reproducibility and unstable conversions [108, 109]. One adverse factor was variable quality of the long and complex RDO molecules, which meant higher doses of reagents and delivery vehicles to effect repair; in turn, these would amplify cytotoxic and proapoptotic actions or induce cell cycle arrest [107, 109]. The problematic nature of these 1st generation reagents was mitigated, however, by findings from the Kmiec group that the all-DNA strand of the RDO initializes the genomic repair [110] and that a single-stranded all-DNA oligonucleotide (ssODN) could outperform the RDO if protected from nuclease degradation by chemical modification of bases [111]. As described in Section 6.3, these data prompted us to switch to short (27-mer) ssODNs, which are purer and give increased reproducibility.

### 6.3. Gene Editing: Use of Single-Stranded All-DNA Oligonucleotides (ssODNs)

The targeting ssODNs are homologous to the genomic sequence except for the desired change—a mismatch to introduce or correct a point mutation, or short insertion/deletion. As single-stranded DNA is rapidly degraded within cells, ssODNs are generally protected with modified nuclease-resistant bases, most commonly three phosphorothioate (PTO) bonds at their 5′ and 3′ ends. Molecular details of targeted gene alteration remain poorly delineated but include elements of nucleotide and base excision repair systems and also some degree of HR involvement [112, 113], while at least part of the correcting DNA oligonucleotide is physically incorporated into the genomic target site [114]. Given the difficulties and controversies associated with RDO-directed repairs, we decided to optimize our methodology in recombinant mammalian cells expressing a sensitive reporter gene, green fluorescent protein (GFP) which was rendered nonfluorescent by introduction of a point mutation. This allowed analysis at the single cell level and the harvesting of corrected (green) cells for further study. We provided stringent evidence to validate the technology and to establish unequivocally that ssODN-mediated gene alteration is a real and reproducible phenomenon [115].

**Figure 3:**
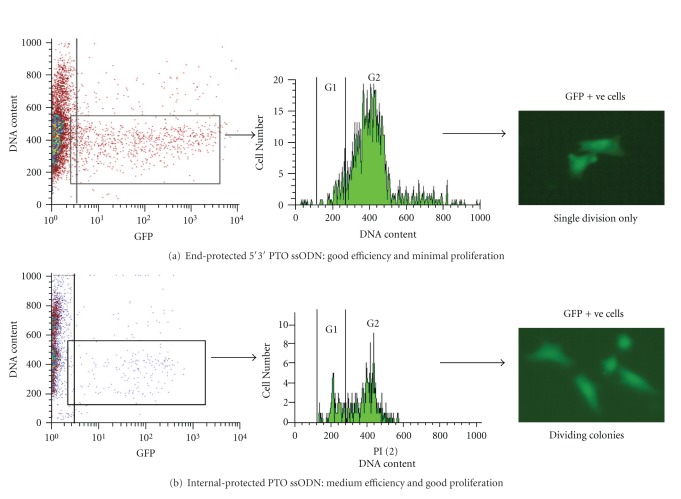
Effects of nuclease-protected ssODNs on targeted gene repair. Recombinant cells expressing mutated (nonfluorescent) green fluorescent protein (GFP) were targeted with two ssODNs, one with three phosphorothioate (PTO) residues at each end (a) and the other with four internal PTOs at the central correcting segment (b). Posttargeting, the green cells were counted by flow cytometry (left panels), stained for cell cycle analysis (central panels) and cultured to observe cell growth (right panels). The PTO end-protected ssODN gave a much higher rate of gene correction, as measured by the number of green cells (boxes in left panels). However, this resulted in DNA damage and accrual of cells in the G2 phase, whereas the GFP+ve cells obtained after internally protected ssODN targeting had a markedly greater proportion in G1 (central panel). Significantly, only the occasional divided pair of green cells was noted in cultures treated with end-protected ssODN, whereas actively replicating green cells were evident when the internally protected ssODN was used (right panels).

Although 5′-3′-PTO protection gave 10-fold greater correction efficiencies than unmodified ssODN (about 2% *versus *0.2% of targeted cells were green), we confirmed the observation of others [111] that only a low percentage of these green cells was actively replicating [116]. Cell cycle analysis 16 h posttransfection revealed that the ssODN with PTO end protection resulted in DNA damage with accumulation of cells in the G2 phase, whereas treatment with unmodified ssODN was markedly less toxic. However, by varying the type or position of protecting groups, we discovered that *internal* protection of the ssODN with four PTO residues at the targeting site maintained efficient gene correction and, compared to 5′-3′-PTO protection, substantially reduced cell cycle arrest to enable cell growth and proliferation (Figure 3) [116].

We are currently evaluating this novel ssODN design in targeting the endogenous *APOE3 *gene in human THP1 monocyte macrophages and human HepG2 hepatoblastoma cells (both *ε*3/*ε*3) to generate cells with the ApoE4 phenotype. We have devised an affinity-capture matrix to distinguish successfully targeted ApoE4-secreting cells (<1%) from noncorrected ApoE3 cells. This uses low permeability carboxymethyl-cellulose media preloaded with a commercial ApoE4-specific monoclonal antibody for detection and gives sensitive and reproducible findings. Secreted ApoE was readily trapped on the surface of individual cells, enhancing staining efficiency and decreasing assay time. Enriched corrected populations of ApoE4-secreting cells have been detected, although isolation and expansion of corrected clones is proving difficult. Should a successful clone be identified and characterized, then it will undergo a 2nd targeting to generate the ApoE4/4 phenotype.

These experiments are notable for two reasons. First, they will provide conclusive proof that ssODN-directed gene editing can generate new cell lines, here, with E3/E4 and E4/4 phenotypes, although a similar strategy can generate the other common phenotypes (E2/E3, E2/2, and E2/4) if an ApoE2-specific antibody is used [117]. Second, because these isogenic cells can be used to investigate isoform differences in macrophage (and hepatocyte) ApoE trafficking and secretion [35, 36], to monitor differential cellular responses to oxidant stress or to cholesterol loading and to investigate variation in structure and composition of secreted ApoE lipoprotein particles [48].

## 7. Therapeutic In Situ Correction of Defective *APOE* Genes

### 7.1. Rationale for ApoE Gene Repair

Most of the in vivo studies described in Sections 2–4 have used ApoE null mice. These gave important insights into ApoE functions and allowed ready evaluation of protein and gene therapeutics. However, as genetic deficiency of ApoE and absent plasma ApoE is extremely rare in humans, the value of such a preclinical model is questionable. It is noteworthy, therefore, that augmentation of plasma ApoE via transgenic expression or gene transfer has confirmed its atheroprotective function in LDLR deficient mice and in diabetic or fat-fed mice, all of which have normal levels of plasma ApoE. Nevertheless, animal studies also indicate caution in augmenting ApoE3 expression using gene transfer technologies; overexpression in mice and rabbits causes hypertriglyceridaemia [19].

An alternative and attractive approach, albeit a distant therapeutic goal, is to alleviate dyslipoproteinaemia and reduce CVD risk by in situ correction of a deleterious *APOE *gene using targeted gene editing. Is this feasible? One possibility, founded on the multiple atheroprotective actions of macrophage-secreted ApoE3, is to cure type III hyperlipoproteinaemia by transplantation of haematopoietic stem cells (HSCs), following ex vivo repair of the aberrant *APOE *gene. As the condition is recessive, conversion of one *ε*2 allele to *ε*3 should provide effective treatment. Moreover, the current impetus in bringing stem cell technologies to the clinic, particularly induced pluripotent stem cells (iPSCs), suggests additional possibilities for ex vivo manipulations and cell-based therapies [118]. These include efficient differentiation to functional hepatocyte-like cells [119–121], which would allow a repaired *APOE *gene to be expressed from cells engrafted into liver. Finally, there is also the exciting prospect of direct in vivo hepatic gene targeting for in situ correction of genetic disease [122]. These sophisticated therapies to repair defective genes in the future will rely on more advanced technologies than simple nuclear delivery of ssODNs and these are discussed in the remaining sections of this paper.

### 7.2. DNA Repair Templates

The ssODNs we have used for targeting of the *APOE* gene have several advantages: high purity and simple production, including variable chemistry, easy delivery, and high fidelity with no off-target effects. Although ssODNs and other constructs such as triple helix forming oligonucleotides and small homologous DNA fragments [113] can act as DNA repair templates, the most effective is adeno-associated virus (AAV), which carries a linear single-stranded DNA genome. Though technically challenging to produce, these viral vectors can genetically target embryonic stem cells (ESCs), HSCs, or iPSCs achieving correction efficiencies of 0.07–1% [113, 123–125]. The mechanism, as illustrated in Figure 5, for targeting and gene repair of defective *APOE2* is via homologous recombination (HR), as silencing essential components of the potentially competitive pathway, nonhomologous end joining (NHEJ) has no effect on conversion frequencies [126]. Note too that the targeting vector can harbour a selectable marker, such as puromycin N-acetyl-transferase, between the homology arms, which if flanked by two LoxP sites can be excised from the genome of repaired cells by Cre-recombinase. More recently, helper-dependent adenoviral vectors (HDAdVs) have also been used for efficient and accurate gene targeting of human ESCs and iPSCs [127, 128]. These vectors are capable of correcting large genomic regions (~35 kb) and are unaffected if the target locus is transcriptionally inactive [129].

### 7.3. Double-Strand Breaks (DSBs) Markedly Stimulate HR Gene Repair

**Figure 4:**
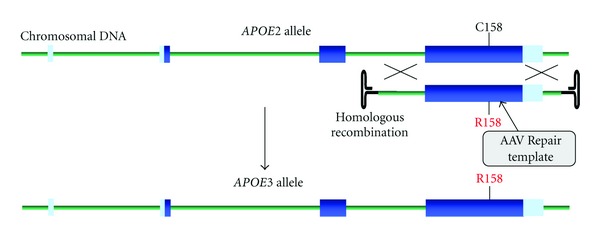
Conversion of the mutant *APOE2 *allele to wild-type *APOE3 *using an AAV-based DNA repair sequence. The single-stranded adeno-associated virus genome, which contains Exon 4 of *APOE3* and its flanking regions within its ITRs (inverted terminal repeats), is an efficient recombination template. As indicated, the mechanism is via a homologous recombination (HR) pathway.

**Figure 5:**
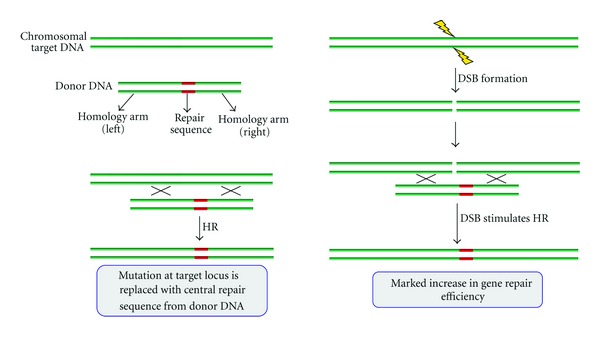
Double-strand breaks (DSBs) stimulate homologous recombination (HR) repair. HR facilitates exchange of DNA sequence between donor and acceptor molecules, provided they share a certain amount of sequence similarity. Donor DNA molecules are designed to contain a central “repair” sequence, flanked by “homology” arms, whose sequence is identical to that of the acceptor (e.g., genomic) DNA. Through HR, the repair sequence replaces the entire sequence lying between the crossover points (marked with an X) on the acceptor. Gene targeting exploits HR in this way to make genetic changes to a cell's DNA. Importantly, introduction of a DSB into the acceptor sequence, which can be achieved by adding an engineered nuclease with locus-specific cleavage (see Figure 6), markedly increases gene repair efficiency.

Although HR-directed gene repair is accurate and versatile, as illustrated in Figures 4 and 5, it occurs with a very low frequency in mammalian cells (~10^−6^) using conventional linear targeting plasmids as templates [130]. However, HR is one way in which cells repair double-strand DNA breaks, for example, by using the sister chromatid as a template, and this phenomenon has been exploited to increase HR gene-targeting events over 1000-fold. Initial studies inserted the rare cutting site for the meganuclease, I-*Sce*I, within a reporter target gene and then cotransfected cells with the endonuclease and a repair DNA template [131, 132]. Nevertheless, such a system has little practical value; it requires prior engineering of the target gene and cannot be used for endogenous genes.

**Figure 6:**
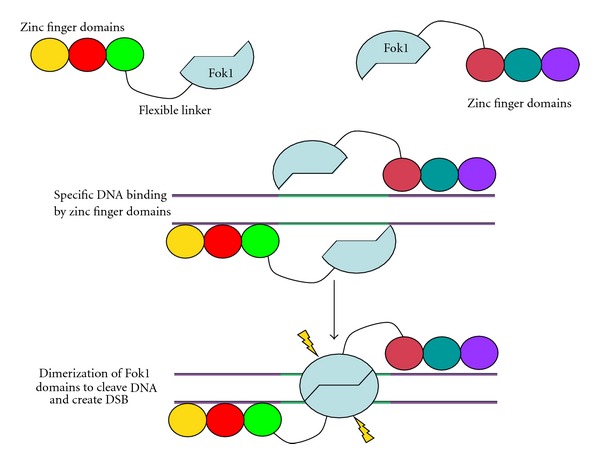
Basic structure and design of a zinc finger nuclease (ZFN). ZFNs are created by joining a DNA-binding region to the catalytic domain of the nonspecific *Fok1* endonuclease. Zinc fingers are a protein motif capable of DNA binding, whose sequence specificity can be predetermined. Each zinc finger, illustrated by an individual circle, recognizes 3-4 nucleotides, and, by assembling three or four suitable zinc finger motifs, a sequence-specific DNA-binding domain can be created. *Fok1* nuclease activity requires dimerization, and so the customized ZFNs function in pairs. As shown, the zinc finger-binding domain brings two *Fok1* units together in the right orientation over the target sequence; this induces *Fok1* dimerization and target sequence cleavage.

Recent work in engineering artificial endonucleases now circumvents this bottleneck by creating chimeric nucleases with the potential to cleave DNA at virtually any desired sequence. The most studied are the zinc finger nucleases (ZFNs), which comprise a DNA-binding domain (assembled as 3–5 finger modules, each recognizing 3 consecutive bases) joined to the nonspecific DNA cleavage enzyme, *FokI*. In principle, customized ZFNs which function in pairs as *FokI* cleavage requires dimerization can be created with predetermined sequence specificity to introduce a DSB at a precise genomic locus (Figure 6). Within the last few years, an impressive number of ZFN gene targeting successes has been reported, including in vitro corrections of 18–30%, the generation of gene-knockout rats, and manipulation of human embryonic or induced pluripotent stem cells [113, 133–136]. One concern of ZFNs is genotoxicity due to off-target cleavages that may disrupt normal genes [137], although improved designs and screening procedures are helping to reduce this possibility [138, 139]. As second problem is that ZFNs can recleave a repaired site, although this can be minimized by introducing a few silent mutations into the donor DNA template as this will impair subsequent ZFN binding [140].

Transcription activator-like effector nucleases (TALENs) are another group of chimeric nucleases, which have a different class of DNA-binding domains coupled to *FokI*. TAL effector proteins contain highly modular DNA-binding domains, with a very simple code between their amino acids and target DNA bases [141, 142]. This allows the ready assembly of a series of individual modules to bind a unique DNA sequence and to activate *FokI *dimerization and cleavage. Although reports of TALEN-mediated gene targeting of mammalian cells are still limited, this simple and effective technology may soon supersede ZFNs; indeed, in the first direct comparison, TALENs had greater specificity and less cytotoxicity than ZFNs [143].

Finally, an alternative to engineered chimeric nucleases are the homing endonucleases, which induce DSBs with exceptional specificity. These are natural enzymes with large asymmetric recognition sequences (12–40 bp) that form five families based on sequence and structure homology [144, 145]. “Mix and match” engineering, aided by computation, allows production and validation of dozens of customized meganucleases, most based on the laglidadg family sequence motif. In turn, prospective DSB sites can be predicted across the entire human genome. This suggests that it will be possible to identify a unique >14-bp sequence within exon 4 of the *APOE *gene that can be cleaved by a homing meganuclease.

### 7.4. ZFN-ssODN Combinations

To date, the most common template for DSB repair-dependent gene targeting has been a circular or linear plasmid, although advantages of using AAV as the exogenous DNA donor substrate are now emerging [146–148]. However, exciting new work establishes that the ZFN-ssODN combination offers flexible and efficient genome editing. Chen and colleagues [149] used ZFNs that cut within the ssODN homology area and reported editing frequencies between 1 and 30% depending on the cell type and whether the targets were single nucleotide substitutions or small to large deletions. Importantly, the ssODN appeared a more efficient and faithful recombination partner than traditional double-stranded DNA constructs, most likely because ssODN does not participate in NHEJ and hence will not insert into nonspecific cleavage sites. Indeed, the duo of ZFN-ssODN has already been used to successfully edit human iPS cells [150]. Given that ssODNs are easier and cheaper to produce in bulk, have high purity and safety profiles, and relatively easy to deliver, it seems likely that their use will continue to expand.

## 8. Concluding Remarks

The technology to repair inborn genetic mutations in cells, including haematopoietic stem cells and induced pluripotent stem cells, has developed rapidly during the last few years. In situ correction of the dysfunctional *ε*2 and *ε*4 alleles to alleviate hyperlipidaemia and counteract the progression of atherosclerosis is now a preclinical reality. It is also translatable to patients. The combination of a selectable AAV-based DNA template for efficient HR repair, which is further stimulated by the safe and precise introduction of a DSB at the target genomic locus, provides the necessary technological tools [146, 147, 151]. Indeed, genome editing using engineered endonucleases was recently announced as *Nature's* Method of the Year for 2011 [152].

Repairing the defective *APOE2 *gene in bone marrow (lineage-negative) stem cells from patients with type III hyperlipoproteinaemia, followed by nonmyeloablative (reduced-intensity) transplantation, will provide macrophage-secreted antiatherogenic ApoE3 at lesions sites and halt atherosclerotic plaque progression. This therapeutic approach can be critically evaluated in a preclinical model, the human ApoE2 knock-in mouse. Moreover, the prospect of direct in vivo hepatic gene targeting for in situ correction of dysfunctional *ε*2 alleles is no longer a distant dream. Codelivery via hepatotropic AAV8 vectors of a chimeric nuclease and donor DNA template to the livers of mice with blood factor IX deficiency restored haemostasis [122].

Is there a case for also converting the *ε*4 allele to *ε*3 to reduce CVD risk when, as outlined in Section 2, the increase may only be marginal even for the *ε*4/*ε*4 carriers? Undoubtedly, the case can be argued: within this group, there will certainly be individuals who would benefit, perhaps because their LDL is in the top decile of *ε*4/*ε*4 carriers or because they have other risk factors. The future prospect of editing the gene in macrophages and/or liver will also allow a more flexible therapeutic approach. Given that the *ε*2 allele is associated with low LDL and reduced CVD, we can also speculate that a better therapeutic option is to convert cells from *ε*4/*ε*4 to the *ε*4/*ε*2 genotype, rather than *ε*4/*ε*3. What is clear, however, is that ApoE, which in 1973 was identified in human VLDL [153], has multiple roles in human cell biology and disease and that, despite impressive understanding of these functions over the last two decades, we still have much more to discover. In time, it is to be hoped that such insights, coupled with the emerging technology of ex vivo or in situ editing of the human *APOE *gene, will eventually translate to patient therapy to reduce CVD risk.

## Abbreviations

AAV: Adenoassociated virus
ABCA1/G1: ATP-binding cassette transporter A1/G1
Apo: Apolipoprotein
CVD: Cardiovascular disease
CETP: Cholesteryl ester transfer protein
DSB: Double-strand break
ESC: Embryonic stem cell
GFP: Green fluorescent protein
HDAdV: Helper-dependent adenovirus
HSPG: Heparan sulphate proteoglycan
HL: Hepatic lipase
HDL: High-density lipoprotein
HR: Homologous recombination
HSC: Haematopoietic stem cell
iPSC: Induced pluripotent stem cell
LCAT: Lecithin-cholesterol acyltransferase
LDL: Low-density lipoprotein
LDLR: LDL receptor
LRP: LDL receptor-related protein
LPS: Lipopolysaccharide
NHEJ: Nonhomologous end joining
PCSK9: Proprotein convertase subtilisin/kexin type 9
PTO: Phosphorothioate
rAdV: Recombinant adenovirus
RDO: RNA-DNA oligonucleotide
scAAV: Self-complementary adeno-associated viral vector
ssODN: Single-stranded oligodeoxyribonucleotide
TALEN: Transcription activator-like nuclease
VLDL: Very-low-density lipoprotein
ZFN: Zinc finger nuclease.
